# Occupational Exposure and Multiple Myeloma Risk: An Updated Review of Meta-Analyses

**DOI:** 10.3390/jcm10184179

**Published:** 2021-09-16

**Authors:** Rebecca Georgakopoulou, Oraianthi Fiste, Theodoros N. Sergentanis, Angeliki Andrikopoulou, Flora Zagouri, Maria Gavriatopoulou, Theodora Psaltopoulou, Efstathios Kastritis, Evangelos Terpos, Meletios A. Dimopoulos

**Affiliations:** Department of Clinical Therapeutics, School of Medicine, National and Kapodistrian University of Athens, 11528 Athens, Greece; rebecca_georg@hotmail.com (R.G.); ofiste@med.uoa.gr (O.F.); tsergentanis@yahoo.gr (T.N.S.); aggandrikop@med.uoa.gr (A.A.); florazagouri@yahoo.co.uk (F.Z.); mariagabria@gmail.com (M.G.); tpsaltop@med.uoa.gr (T.P.); ekastritis@gmail.com (E.K.); mdimop@med.uoa.gr (M.A.D.)

**Keywords:** multiple myeloma, occupation, occupational hazards, risk factors, occupational epidemiology

## Abstract

The precise etiology of multiple myeloma remains elusive, but both genetic and environmental factors have been suggested to contribute to disease risk. Several occupational categories and toxic agents have been implicated as potentially causative, yet findings from the literature are inconsistent. The aim of this review was to summarize and critically comment on the accumulated epidemiological evidence, across published meta-analyses, about the association between occupational exposure and risk of multiple myeloma. Overall, results from eleven meta-epidemiological studies underscore a significantly increased risk for firefighters, hairdressers, and employees exposed to engine exhaust, whereas farming and methylene chloride exposure have been non-significantly correlated with the disease. Further epidemiological studies are of utmost importance whilst emphasis should be placed on occupational hazard surveillance, as such studies will obtain a more accurate picture of disease occurrence in working populations, and will enable both the implementation of preventive actions and the evaluation of their effectiveness.

## 1. Introduction

Multiple myeloma (MM), a proliferative disease of immunoglobulin-secreting mature B cells, known as plasma cells, is the second most frequent hematologic malignancy, accounting for approximately 13% of neoplastic diseases of the blood and 1% of all cancers [1,2,3,4]. In 2018, there were about 160,000 cases of MM, translating to an age-standardized incidence rate of 1.8 per 100,000 persons, while the overall survival has been greatly improved over the past decade with the advances in treatment modalities, with an overall 5-year survival rate of 54% [5,6,7].

Although the precise etiology of the disease has not yet been established, the asymptomatic, premalignant monoclonal gammopathy of undetermined significance (MGUS) and smoldering multiple myeloma (SMM) are thought to be precursor states of MM [8,9], whereas male sex, older age, African American ancestry, genetic susceptibility, and obesity have been acknowledged as risk factors [10,11,12,13].

Environmental epidemiology of MM is an increasingly investigated, yet controversial, field. Numerous systematic reviews and meta-analyses for multiple environmental and occupational risk factors associated with MM have been published. Thus far, a systematic review of meta-analyses conducted by Sergentanis T. N. et al. [14] in 2015 examined a wide variety of risk factors for MM, including occupational exposure. As new results from incidence and mortality studies have become available since this systematic review [14], and given the significance of meta-analyses as powerful quantitative tools of occupational health policy [15], we have conducted an updated review of published meta-analyses, in order to provide an overview of the range and validity of the reported associations of diverse occupational risk factors with MM.

## 2. Materials and Methods

### 2.1. Search Strategy and Eligibility Criteria

A comprehensive review of published literature was conducted to evaluate associations between occupational risk factors and MM. Eligible studies were systematically sought in MEDLINE/PubMed database up to 31 July 2021. Relevant keywords for the search algorithm were (myeloma OR ‘’multiple myeloma’’) AND (occupation OR ‘’occupational factors’’) AND (meta-analysis OR meta-analyses).

The full text of potentially eligible articles was scrutinized by two authors (RG and OF), who worked independently and blindly to each other. Eligible studies included meta-analyses examining the contribution of the workplace environment to MM. We excluded meta-analyses that investigated occupational risk factors for other medical conditions including other hematological malignancies, meta-analyses that examined non-occupational risk factors for MM, and meta-analyses of therapeutic regimens for MM. Furthermore, reviews, systematic reviews, and pooled analyses were also excluded. We did not apply any language restrictions in the selection of eligible studies.

### 2.2. Data Extraction

Data extraction and analysis was done independently by two investigators (O.F. and R.G.), and in the case of inconsistencies and/or disagreements the final decision was reached by team consensus. From each eligible meta-analysis, we extracted information on the first author, journal and year of publication, examined risk factors, and number of studies included. We also extracted the number of cases and controls included, the study-specific effect size measure (i.e., risk ratio (RR), odds ratio (OR), hazard ratio (HR)) together with their corresponding confidence interval (CI), the *p* value (and/or I^2^) for heterogeneity, and publication bias.

## 3. Results

From the 37 articles retrieved from our search strategy, nine were deemed irrelevant from their abstracts. From the remaining 28 articles, four were pooled analyses, six were systematic reviews or reviews, one was a retrospective national cohort study, and one evaluated the association between MM incidences and residential exposure to the petrochemical industry. Overall, 11 meta-analyses [16,17,18,19,20,21,22,23,24,25,26] providing results from 165 primary studies of potential occupational risk factors were ultimately eligible for this review, since five [27,28,29,30,31] have been updated by more recent meta-epidemiological studies. Characteristics of the included studies are presented in Table 1.

### 3.1. Agriculture and Farming

Blair A. et al. [27] conducted the first meta-analysis on cancer risk among farmers and found that this occupational group had a significantly elevated risk for MM (OR: 1.12; 95% CI: 1.04–1.21). In 1997, an updated meta-analysis by Khuder S. A. and Mutgi A. B. [16], incorporating results from 32 studies published between 1981 and 1996, confirmed the positive association between farming and MM (RR: 1.23; 95% CI: 1.14–1.32).

The risk of hematologic malignancies in pesticide-related occupations was evaluated by Merhi M. et al. [28]. In their meta-analysis, among a subset of 13 case–control studies, two of them were restricted to MM risk. They concluded that the use of pesticides in occupational activities may increase MM risk, but this was not statistically significant (pooled OR: 1.16; 95% CI: 0.99–1.36; *p* = 0.06).

Perrotta C. et al. [17] also confirmed the increased risk in developing MM for farmers (OR: 1.39; 95% CI: 1.18–1.65) and for those working with pesticides (OR: 1.47; 95% CI: 1.11–1.94), in a meta-analysis of 28 case–control studies, with the limitations of significant heterogeneity across the studies and publication bias in some models. Recently, a meta-analysis of one cohort and two case–control studies [18] revealed a lack of association between the exposure to glyphosate, a broad-spectrum systemic herbicide and crop desiccant, and MM risk (meta-RR: 1.04; 95% CI: 0.67–1.41; *p* = 0.21).

### 3.2. Firefighting

A meta-analysis of eight mortality studies in 2006 [29] revealed an elevated pooled relative risk for MM among firefighters (Summary Risk Estimate (SRE): 1.53; 95% CI: 1.21–1.94). Similar significant and consistent findings were also identified in the updated meta-analysis by Soteriades E. S. et al. [19]; the relative risk estimate for mortality was 1.28 (95% CI: 1.03–1.58; *p* < 0.05). Potential causative compounds include benzene, PAHs, aldehydes, and other organic chemicals.

### 3.3. Hairdressing and Allied Occupations

Takkouche B. et al. [20] found that across 19 studies, hairdresser occupation increases the risk of MM by 62% (the fixed-effects RR: 1.38; 95% CI: 1.25–1.54; the random-effects RR: 1.62; 95% CI: 1.22–2.14; *p* = 0.0001). Frequently used hazardous chemicals among hairdressers include formaldehyde, ammonium compounds, polyvinylpyrrolidone, and organic solvents.

### 3.4. Organic Solvents

The meta-analysis of case–control studies conducted by Sonoda T. et al. [21] indicated a statistically significant positive association between MM risk and engine exhaust exposure (OR: 1.34; 95% CI: 1.14–1.57), but failed to identify significant associations for petroleum, petroleum products, and benzene. On the contrary, in a meta-analysis of seven cohort studies, Infante P. F. [30] demonstrated a statistically significant correlation between benzene exposure and risk of death from MM (RR: 2.13; 95% CI: 1.31–3.46). The updated meta-analysis by Vlaanderen J. et al. [22], synthesizing 26 cohort studies, revealed a slight, nonsignificant elevation of the overall meta-RR for those who experience occupational benzene exposure (meta-RR: 1.12; 95% CI: 0.98–1.27). Recently, Onyije F. M. et al. [23], in a meta-analysis consisting of 11 cohort studies and one case–control study, demonstrated consistent positive findings regarding petroleum industry work and incidence of MM (estimated risk of 1.80; 95% CI: 1.28–2.55), yet failed to support significant correlation between petroleum exposure and risk of mortality from MM (estimated risk of 1.04; 95% CI: 0.89–1.21).

The results from a meta-analysis of seven cohort studies [31] did not support associations between occupational TCE exposure and MM risk (pooled RR: 1.05; 95% CI: 0.80–1.38; *p*-heterogeneity: 0.94). Consistent findings had been revealed by a more recent study of Karami S. et al. [24]; meta-analytical results of nine cohort and two case–control studies failed to report significant associations between TCE exposure and MM (RR: 1.05; 95% CI: 0.88–1.27; *p*-heterogeneity: 0.76).

Regarding exposure to methylene chloride and MM risk, the meta-analysis of Liu T. et al. [25] included one case–control and two cohort studies and pointed to a positive, significant association (the fixed-effects OR: 2.04; 95% CI: 1.31–3.17) without evidence of heterogeneity among studies. Both mechanistic and epidemiological studies are ultimately warranted to provide insights into the carcinogenic potential of methylene chloride, and the realistic risk of hematopoietic cancer in particular.

### 3.5. Other Occupational Factors

Alicandro G. et al. [26] conducted a meta-analysis of 11 cohort studies examining the risk of lymphatic and hematopoietic neoplasms among workers exposed to polycyclic aromatic hydrocarbons (PAHs). Meta-analytic estimates revealed a nonsignificant excess risk of MM (meta-RR: 1.18; 95% CI: 0.93–1.50) among workers in aluminum production. On the contrary, no associations were found between MM and occupational exposure for iron and steel foundry workers (meta-RR: 1; 95% CI: 0.67–1.51; *p* = 0.26) and occupational exposure for asphalt workers (meta-RR: 0.72; 95% CI: 0.42–1.23; *p* = 0.77). Overall, at the sample size of the included studies, a positive correlation between PAHs exposure and MM risk could not be identified, with the effect size commensurate with the power of the studies.

## 4. Discussion

To date, results from epidemiological studies of potential risk for MM mediated by occupation exposure have been inconsistent. In particular, several risk factors across five categories, including farming, firefighting, hairdressing, and organic solvents and PAHs exposure have been studied for an underlying causal association with MM. Herein, we provided an overview and appraisal of the occupational epidemiology of MM, presenting data from published meta-epidemiological studies. Since publication of the previous systematic review of meta-analyses by Sergentanis T. N. et al. [14], four new meta-analyses of occupational exposures and MM were considered in our updated review [18,19,23,26]; nonsignificant associations between glyphosate [18], PAHs [26], and petroleum [23] exposure and MM risk have been reported, whereas, consistent with previous findings [29], firefighting correlated with increased risk of death from MM [19]. Overall, the results from the included meta-epidemiological studies [16,17,18,19,20,21,22,23,24,25,26] in this updated review confirm the statistically significant risk for MM among firefighters, hairdressers, and employees exposed to engine exhaust, highlighting the multifactorial traits of the disease.

Findings from studies of farming-related occupations and MM risk have been inconsistent, ranging from positive associations [32,33,34,35,36,37,38,39] to inverse associations [27,40,41,42,43,44,45,46,47,48,49,50]. In a large pooled analysis of five international case–control studies [51] including 1959 MM cases and 6192 control subjects over a period of 30 years, gardeners and nursery workers possibly exposed to pesticides, showed a 50% increase in risk (OR:1.50; 95% CI: 0.9–2.3) while other agricultural jobs did not. With regard to published meta-analyses, as presented in our review, Khuder S. A. et al. [16] and Perrotta C. et al. [17] highlighted farming as a risk factor, whereas Donato F. et al. [18] did not find significant associations between MM and pesticide and herbicide exposure, respectively, which could be attributed to the relatively small sample size, and thus, to inadequate statistical power.

Moreover, we should also address that in agriculture the range of occupational exposure is quite broad, considering that farmers may use a number of hazardous products, including pesticides, herbicides, engine fuels and exhausts, fertilizers, and other chemical solvents [52,53]. As a result, the heterogeneity of both type and dose level of exposure, in addition with the variety in intensity of exposure (i.e., duration of working time or seasonal application of pesticides and herbicides) could partially explain the observed inconsistency across published studies, and thus should not be underestimated.

With regard to firefighting, in a pooled cohort of 30,000 US firefighters, Daniels R. D. et al. [54] reported increased cancer incidence among this occupational group, in comparison with the general population. Soteriades E. S. et al. [19] highlighted the increased risk of MM among firefighters. Indeed, during firefighting activities, firefighters may be exposed (via inhalation and/or dermal absorption) to both known and suspected carcinogens, including acetaldehyde, formaldehyde, the non-threshold toxicant benzene, PAHs, asbestos, cadmium, and arsenic [29,55,56,57,58,59]. Given the varied exposure and emissions levels, the quantification of relative myeloma risk remains quite challenging.

Employees in hairdressing salons, barbershops, and beauty salons are also exposed to a variety of agents released from hairdressing and beauty products, like hydrogen peroxide, ammonia, formaldehyde, nitrosamines, polyvinylpyrrolidone/polyvinyl-acetate copolymer (PVP-PVA), some of which have been suggested as mutagenic or carcinogenic [60]. In a large meta-analysis, with no evidence of publication bias, but with high degree heterogeneity among included studies, Takkouche B. et al. [20] found a significant positive correlation between MM and the hairdresser’s profession. Despite usually being short-term, the repetitive airborne and dermal exposure to certain hazardous chemicals, and especially organic solvents, could explain the elevated MM risk in this occupational cohort.

Benzene, one of the elementary petrochemicals widely used for the production of polymers, plastics, rubbers, dyes, pesticides, lubricants, and as a component of unleaded gasoline, has been associated with hematopoietic cancer, including MM [61]. The first meta-analysis, which included only case–control studies [21], failed to detect any significant correlation between occupational benzene and petroleum exposure with MM, but suggested a significant positive association with engine exhaust exposure. Similarly, a recent meta-analysis comprising one case–control and 11 cohort studies [23] noted increased risk for MM among petroleum industry workers.

Additionally, no supportive evidence for increased MM risk following occupational exposure to TCE [23] and PAHs [25] could be identified, while meta-analytical results yielded positive associations of methylene chloride exposure and MM [25].

Our study has several caveats, which we should critically point out as they reflect key limitations of meta-analyses. Firstly, given the relative rarity of MM, cohort studies may lack adequate statistical power, while case–control studies may suffer from small sample sizes for specific occupational categories [62,63]. Moreover, in case–control studies, selective enrolment of participants as controls could introduce selection bias, whereas differential recall, between cases and controls, of information on exposure depending on their outcome could introduce recall bias [62]. In addition, the presence of confounding factors, the exposure to a variety of chemical agents in agricultural work environments, and limitations of used statistical methods could blur the results of occupational epidemiological studies [64]. Additionally, both studies’ heterogeneity and publication bias represent challenges in the interpretation of meta-analyses [64].

Furthermore, we need to address that overlapping data between specific studies may occur, leading to spurious associations and false positive results. Of note, despite the fact that 14 studies [65,66,67,68,69,70,71,72,73,74,75,76,77,78] included in the meta-analysis by Khuder S. A. et al. [16] were also included in the meta-analysis by Perrotta C. et al. [17] (Table 2), they were both retained because the second comprised of only case–control studies, while the first also contained two cohort studies. Lastly, it is evident from Table 1 that some of the included meta-analyses were of low methodological quality, given the poorly reported essential elements of their study design or results, thus diminishing their value to clinicians and policy makers.

Moreover, the identification of specific occupational categories related with an increased risk of MM enables public health officials to not only identify populations (i.e., with plasma cell precursor conditions like MGUS and SMM) in need of earlier and more frequent screening tests (i.e., serum and urine protein electrophoresis), but also implement feasible and effective preventive measures. Thus, large-scale epidemiological studies of high quality are warranted to investigate and further characterize potential workplace hazards related with MM.

## 5. Conclusions

In summary, the present review focused on the published meta-analyses that summarize current knowledge on occupational risk factors for MM epidemiology. Additional evidence from well-designed epidemiological studies in the near future is anticipated to further shed light on repeatedly reported associations of MM risk with various occupational risk factors.

## Figures and Tables

**Table 1:** Characteristics of included meta-analyses.

Author (Publication Year)	Risk Factor	Number of Primary Studies	Total Number of Cases/Total Number of Controls and/or Exposed Cases and/or Unexposed Cases	Effect Size Metric	Pooled Effect Size (95% CI)	I^2^ (%)	*p*-Value	*p*-Heterogeneity	Main Results	Publication Bias
Khuder S. A. et al. (1997) [16]	Farming	32 studies (19 case–control studies, 5 PMR, 4 SIR, 2 SMR, 2 cohort studies)	4165 exposed cases/NA	RR	1.23 (1.14–1.32). The estimator of RR obtained from a meta-analysis restricted to female farmers was 1.23 (1.17–1.29)	NA	NA	NA	Positive association between MM and farming	No evidence of publication bias
Perrotta C. et al. (2008) [17]	Farming (pesticide and herbicide exposure)	28 case–control studies	NA	OR	Farming: OR = 1.39 (1.18–1.65)	Significant heterogeneity across the studies	NA	0.002	Farmers seem to have increased risk for MM. Exposure to pesticides seems to be a possible risk factor.	NA
					Pesticide exposure: OR = 1.47 (1.11–1.94)	Evidence of heterogeneity across the studies	NA	0.09		Evidence of publication bias
					Herbicide exposure: OR = 0.97 (0.68–1.38)	NA	NA	NA		NA
Donato F. et al. (2020) [18]	Glyphosate (herbicide)	3 studies (2 case–control studies, 1 cohort study)	290 exposed cases/NA	meta-RR	1.04 (0.67–1.41)	16%	NA	*p* = 0.21	No consistent indication of an association between exposure to glyphosate and risk of MM	NA
Soteriades E. et al. (2019) [19]	Firefighting	8 studies	NA	Risk estimate for mortality	1.28 (1.03–1.58)	NA	*p* < 0.05	NA	For MM the authors found statistically significant association with firefighting	NA
Takkouche B. et al. (2009) [20]	Hairdresser occupation	19 studies (8 case–control studies, 3 PMR, 8 cohort studies)	17,567 cases/68,301 controls (of all hematologic cancers)	RR	Fixed-effects RR: 1.38 (1.25–1.54). Random-effects RR: 1.62 (1.22–2.14)	0.75	*p* = 0.0001	NA	Substantial MM risk among employees of the hairdressing industry	No evidence of publication bias
Sonoda T. et al. (2001) [21]	Benzene exposure	Benzene and/or organic solvents: 8 case–control studies	15,614 cases/75,054 controls	OR	0.74 (0.60–0.90)	NA	Significantly decreased	NA	Significant positive association between exposure to engine exhaust and MM. No significant associations between MM and benzene and/or organic solvents, petroleum, and petroleum products.	NA
		Petroleum: 6 case–control studies	3873 cases/12,250 controls		1.11 (0.96–1.28)		Not significant	NA		
		Petroleum products (rubber and/or plastic products): 7 case–control studies	27,925 cases/133,486 controls		1.08 (0.89–1.33)		Not significant	NA		
		Engine exhaust: 7 case–control studies	4750 cases/14,580 controls		1.34 (1.14–1.570)		Statistically significantly elevated	NA		
Vlaanderen J. et al. (2011) [22]	Benzene exposure	26 cohort studies	284 cases/NA	meta-RR	1.12 (0.98–1.27)	NA	*p* = 0.35	NA	Nonsignificant association between benzene exposure and MM	Evidence of publication bias
Onyije F. M. et al. (2021) [23]	Benzene exposure	12 studies (5 SIR, 9 SMR studies)	NA	Risk estimate for incidence and mortality	1.80 (1.28–2.55) for incidence; 1.04 (0.89–1.21) for mortality	0% (for incidence); 16% (for mortality)	NA	*p* = 0.81 (for incidence); *p* = 0.30 (for mortality)	Increased risk for both incidence and mortality between petroleum exposure and MM	No evidence of publication bias
Karami S. et al. (2015) [24]	Trichloroethylene (TCE) exposure	11 studies (9 cohort studies, 2 case–control studies)	114 TCE-exposed cases of MM out of 273,423 subjects (cohort studies). 75 cases and 255 TCE-exposed controls (case–control studies)	RR	1.05 (0.88–1.27)	6.69	NA	*p* = 0.76	Meta-analytical results for cohort and case–control studies did not show significant associations between occupational TCE exposure and MM	No evidence of publication bias
Liu T. et al. (2013) [25]	Methylene chloride exposure	3 studies (1 case–control study, 2 cohort studies)	NA	OR	2.04 (1.31–3.17)	0%	*p* > 0.1	*p* = 0.871	Supportive results of a positive significant association of methylene chloride exposure and MM	NA
Alicandro G. et al. (2016) [26]	Polycyclic aromatic hydrocarbons (PAH)	Aluminum production: 5 cohort studies	68/39,241	meta-RR	1.18 (0.93–1.50)	0%	NA	*p* = 0.34	Occupational PAH exposure does not associate with a significant excess risk of MM	No evidence of publication bias
		Iron and steel foundry: 4 cohort studies	23/23,145		1 (0.67–1.51)	0%	NA	*p* = 0.26		
		Asphalt workers: 2 cohort studies	13/30,686		0.72 (0.42–1.23)	0%	NA	*p* = 0.77		
					High exposure level: 0.96 (0.73–1.27)		Not significant			

Abbreviations: CI = confidence interval; HR = hazard ratio; meta-RR = meta-relative risk; MM = multiple myeloma; NA = not available; OR = odds ratio; PMR = proportionate mortality ratio; RR = relative risk; SIR = standardized incidence ratio; SMR = standardized mortality ratio; SRE = summary risk estimate; SRRE = summary relative risk estimate.

**Table 2:** Overlapping studies from the meta-analyses by Khuder S. A. et al. and Perrotta C. et al.

Author (Publication Year)	Type of Case–Control Study	Total Number of Exposed Cases	Relative Risk (95% CI)
Gallagher et al. (1983) [65]	Incident	31	2.20 (1.20–4.00)
Cantor and Blair (1984) [66]	Mortality	175	1.40 (1.00–1.80)
Nandakumar et al. (1986) [67]	Mortality	21	1.44 (0.81–2.55)
Pearce et al. (1986) [68]	Incident	43	1.70 (1.00–2.90)
Flodin et al. (1987) [69]	Incident	30	1.90 (1.10–3.10)
Cuzick and De Stavola (1988) [70]	Incident	28	1.60 (0.87–2.94)
Brownson et al. (1988) [71]	Incident	24	1.40 (0.87–2.24)
Boffetta et al. (1989) [72]	Incident	16	3.40 (1.50–7.50)
La Vecchia et al. (1989) [73]	Incident	25	1.90 (1.10–3.20)
Heineman et al. (1992) [74]	Incident	45	1.10 (0.80–1.50)
Eriksson and Karlsson (1992) [75]	Incident	151	1.68 (1.16–2.44)
Blair et al. (1993) [76]	Mortality	489	1.13 (1.03–1.24)
Demers et al. (1993) [77]	Incident	26	1.20 (0.80–2.50)
Francheschi et al. (1993) [78]	Mortality	20	1.30 (0.70–2.30)

Abbreviations: CI = confidence interval.
